# Multi-Generational Review of Oncologic Tumors in a Family With TP53 Mutation Presenting With a Pediatric Patient With Osteosarcoma and Lung Acinar Adenocarcinoma

**DOI:** 10.7759/cureus.17271

**Published:** 2021-08-18

**Authors:** Henna Butt, Ashley Munchel, Teresa York, Regina Macatangay

**Affiliations:** 1 Pediatrics, University of Maryland Medical Center, Baltimore, USA; 2 Pediatric Oncology, University of Maryland Medical Center, Baltimore, USA

**Keywords:** tp53, li-fraumeni, toronto protocol, acinar adenocarcinoma, osteosarcoma

## Abstract

TP53 mutation, Li-Fraumeni syndrome (LFS), is a syndrome that leads to a hereditary cancer predisposition. Here we describe the case of a 13-year-old male who presented with osteosarcoma, family history of LFS, who developed a second primary tumor of the lung. No other similar cases have been reported. After this osteosarcoma diagnosis, he had pre-operative imaging, which included a positron emission tomography (PET) combined with CT (PET/CT) chest. This revealed a subpleural nodule in the lung of unclear etiology. After completing initial therapy, a repeat chest CT showed that the nodule persisted. Pathology revealed an acinar adenocarcinoma. This tumor is not common in pediatric LFS patients.

## Introduction

TP53 gene mutation, also known as Li-Fraumeni syndrome (LFS), is an autosomal dominant syndrome that leads to a hereditary cancer predisposition [1]. First described in 1969 [2], it is a rare condition and affects around one in every 5,000 to 20,000 people worldwide [1]. This syndrome results in the diagnosis of a variety of tumor types at a young age, with a characteristic family clustering pattern. It can involve multiple primary tumors and is associated with a broad spectrum of tumors distinguishing it from other cancer syndromes. The core spectrum of cancers includes bone and soft-tissue sarcomas, CNS tumors, leukemia, adrenocortical carcinoma, and breast cancer. Most diagnoses are made prior to 10 years of age, followed by a second peak between 30 and 50 years of age. Diagnosis is made based on molecular diagnosis involving the TP53 gene [1]. P53 is a transcription factor that functions to protect cellular mechanisms against stressors and acts as a tumor suppressor. Loss of this protective gene leaves patients susceptible to a range of solid and hematologic cancers [2]. Genetic testing is usually prompted when a patient presents with a tumor strongly associated with LFS. A thorough analysis of the family history should be done, including a minimum of three generations. Gold standard testing involves the detection of pathogenic mutations by sequencing the entire TP53 coding region [1]. Here we present the case of a 13-year-old male who presented with humeral osteosarcoma, known family history of LFS, who subsequently developed a second primary tumor of the lung. Upon the literature review, no other cases of similar presentation have been reported. This case has been presented at the American Society of Pediatric Hematology/Oncology Conference 2021.

## Case presentation

**Figure 1:**
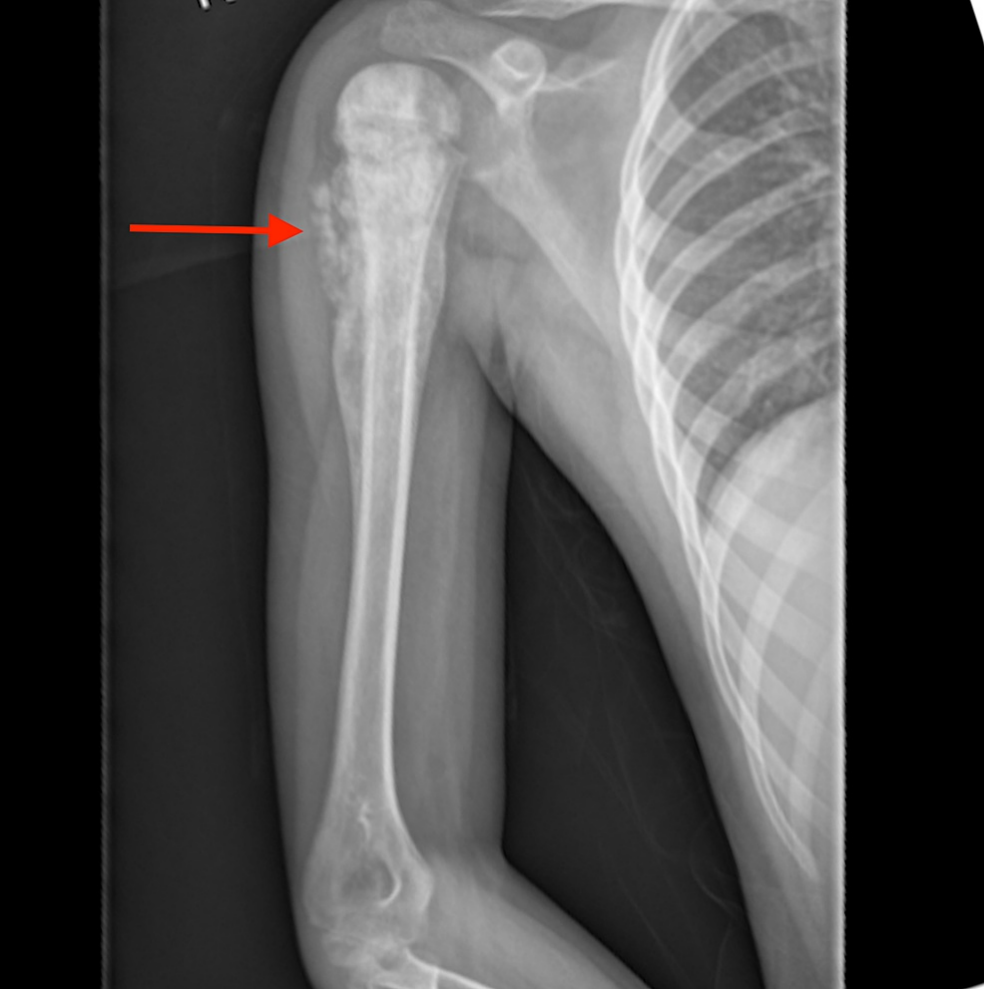
Anterior-Posterior (AP) radiograph depicts the classic periosteal elevation resulting in the Codman triangle. It also highlights the periosteal reaction resulting in cloud-like bone formation. The tumor seen involves the metaphyseal region of the humerus with extension into the diaphysis. There is also a wide zone of transition which has been shown to be a feature of aggressive lesions.

**Figure 2:**
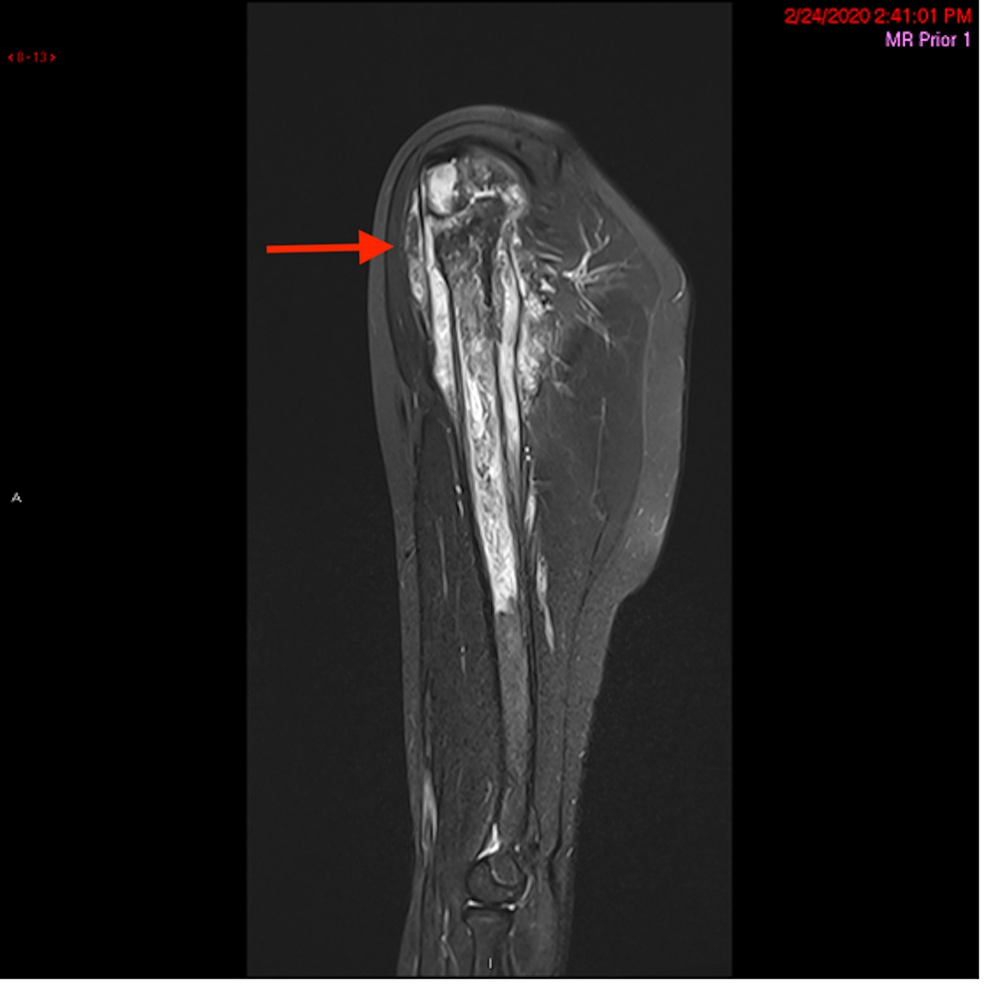
MRI of the right upper extremity. Sagittal fluid sensitive sequence with fat saturation shows increased T2 signal involving the epiphysis, metaphysis, and diaphysis of the proximal humerus. Note the subperiosteal soft tissue extension and adjacent muscle edema (red arrow).

**Figure 3:**
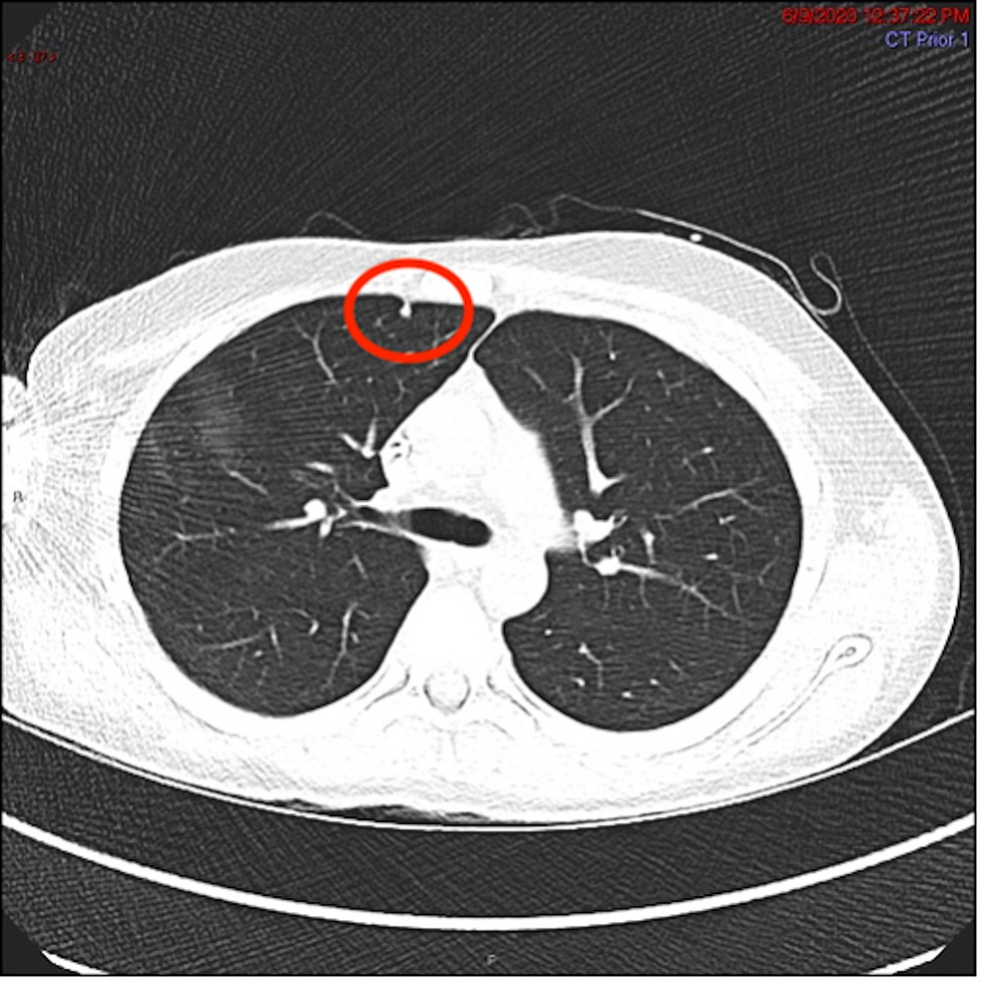
CT chest. Axial lung windows show a 5 mm pleural-based lung nodule in the anterior right upper lobe (red circle).

The patient is a male who presented at the age of 12 to the Pediatric Oncology Practice for persistent right upper arm pain for six weeks. Due to progressive edema, an X-ray was performed which revealed a right humeral lesion (Figures 1-2). The patient underwent an open biopsy of the right proximal humerus. Diagnostic evaluations confirmed the diagnosis of osteosarcoma. Prior to surgical resection, pre-op imaging with positron emission tomography (PET) combined with CT (PET/CT) revealed a 5 mm subpleural nodule in the right upper lobe of unclear etiology (Figure 3). This lesion was monitored with serial imaging to determine if chemotherapy would have an effect on size. 

Ten weeks following neoadjuvant chemotherapy with cisplatin, doxorubicin, and high-dose methotrexate, our patient had a radical resection of his right proximal humerus. One month later, he required further surgeries due to postoperative wound complications. After completing 21 weeks of chemotherapy, a repeat CT chest showed that the subpleural nodule did not demonstrate treatment effect and remained at 5 mm. After a multidisciplinary discussion involving orthopedic surgery, pediatric oncology, and radiology, the decision was made to obtain a thoracoscopic biopsy of the right upper lobe to rule out metastatic osteosarcoma. Pathology revealed an acinar adenocarcinoma with a positive margin. He underwent wedge resection of the lesion with no residual malignancy. 

**Figure 4:**
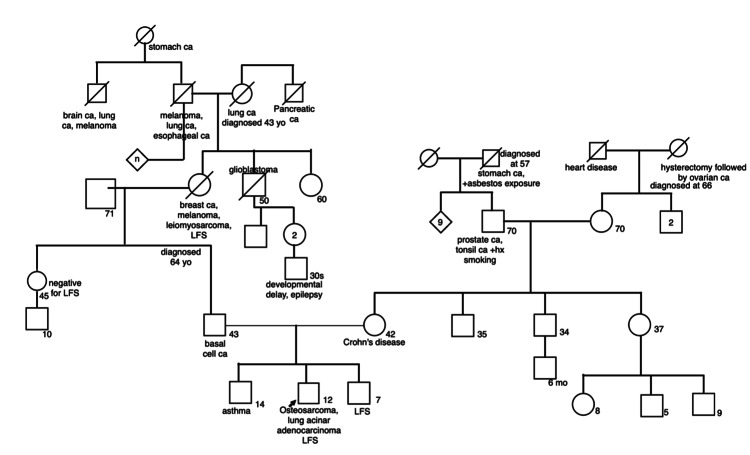
Pedigree of family described in this case. ca: Carcinoma; yo: Years old; hx: History; mo: Months old.

Prior to the presentation, the patient’s parents were aware that there was a family history of LFS. During this patient’s first admission for chemotherapy, genetics was consulted, given his family history of LFS. See full pedigree below (Figure 4). His paternal grandmother had breast cancer, leiomyosarcoma, melanoma and was found to have LFS. Her testing identified a heterozygous c.636deIT (p.R213fs) deletion mutation in the TP53 gene. His paternal grandmother’s brother died of brain cancer and his paternal great-grandmother died of lung cancer. His father tested positive for the same TP53 mutation and his younger brother was found to have LFS during screening. Molecular analysis of the proband’s DNA identified heterozygous c.473G>A (pR158H) pathogenic variant in the TP53 gene. His brother was noted to have the same mutation.

## Discussion

Patients with LFS have a very high lifetime cumulative risk of developing multiple malignancies and a strong family history of early-onset malignancies. Soft tissue sarcomas, osteosarcomas, premenopausal breast cancer, brain tumors, and adrenal cortical carcinomas are all LFS-associated tumors. As can be seen in the pedigree of this family, there is an abundance of tumors that can be seen in families affected by LFS. His paternal grandmother developed multiple malignancies that are typical of LFS. This patient’s pedigree shows a classic autosomal dominant inheritance pattern, and while it cannot be confirmed by genetic analysis at this time, it is likely that earlier generations may have had carriers of LFS based on his pedigree. Also, important to note is that his father is currently undergoing surveillance for cancer detection. With regards to his younger brother, who has also tested positive for TP53 mutation, he is currently undergoing surveillance imaging per the Toronto Protocol. 

The Toronto Protocol is a multi-modality protocol that was developed by investigators in Toronto, Salt Lake City, Los Angeles, and Columbus. The goal was to detect tumors early and reduce morbidity and mortality in patients who are TP53 mutation carriers. They found an improved overall survival of 88.8% in five years compared to 59.6% in patients who did not undergo surveillance. This protocol includes complete physical exams and then groups modalities under specific categories of tumors. The different modalities include ultrasound, MRI, colonoscopy, and esophagogastroduodenoscopy (EGD). These screening modalities change depending on the age and sex of the patient, as certain tumor incidences vary based on these factors [2].

Whole-body MRI is becoming more widely used given its utility in assessing multiple organ systems, high sensitivity, and lack of ionizing radiation. Children with LFS have increased radiosensitivity, making CT less suitable for long-term surveillance. For this reason, an annual whole-body MRI from the time of diagnosis is recommended. Whole-body MRI has an NPV of 100% in pediatric cancer predisposition syndrome patients [3].

Our patient underwent a CT chest to further examine the lung nodule found in earlier imaging. Despite the risks of ionizing radiation, the CT chest is currently the best modality in detecting lung nodules. Findings seen in Figure 2 will impact his treatment course and led to the diagnosis of his lung adenocarcinoma. Further research for imaging techniques is needed for cancers that are not commonly seen in patients with TP53 mutation. Currently, the surveillance protocols published do not include routine screening modalities for lung cancers [2]. 

Typically, osteosarcomas in LFS patients occur in the ages between 10 and 14 years and are slightly more frequent in males [4]. Our case fits this profile. While his osteosarcoma presentation is typical given his LFS diagnosis, the development of his lung acinar adenocarcinoma is not common. One case was reported in a 26-year-old female with a hereditary familial overlap syndrome (LFS plus CHD1) who developed multiple synchronous primary lung adenocarcinomas several years after diagnosis of osteosarcoma. She completed chemotherapy for osteosarcoma and then was found to have a nodule in her lung at 24 years of age. Pathology confirmed acinar well-differentiated lung adenocarcinoma [5]. The authors of that case report hypothesized that the development of her multiple tumors was a result of her having both gene mutations. A study conducted by Birch JM et al. looked at the spectrum and frequency of cancers associated with germline TP53 mutations. Using a cohort of individuals from 28 families with LFS, they were able to characterize that certain cancer types were strongly associated with TP53 mutations, some were moderately associated and others were weakly associated. When specifically looking at lung cancer, they found that there was no significant association with germline TP53 mutation [6]. Based on current literature, given our patient’s age and diagnosis of acinar lung adenocarcinoma, it is clear that his presentation is unique despite his diagnosis of LFS.

## Conclusions

This patient was confirmed to have TP53 mutation, which is also known as LFS, an autosomal dominant cancer predisposition syndrome. It involves multiple primary tumors and is associated with a broad spectrum of hematologic and solid malignancies. Current surveillance protocols do not include routine screening modalities for lung cancers. This brings to light that the risks and benefits of surveillance imaging are needed to be considered further in LFS patients. Despite the risks of ionizing radiation, the CT chest is currently the best modality in detecting lung nodules. Findings from his chest CT will impact his treatment course and led to the diagnosis of his lung adenocarcinoma. For this reason, further research on imaging techniques is needed for cancers that are not commonly seen in patients with TP53 mutation.
